# Gastrointestinal function and microbiota in endurance athletes

**DOI:** 10.3389/fphys.2025.1551284

**Published:** 2025-05-14

**Authors:** Daichi Sumi, Yoshio Suzuki

**Affiliations:** ^1^ Institute of Sport Science, ASICS Corporation, Kobe, Japan; ^2^ Graduate School of Health and Sports Science, Juntendo University, Inzai, Chiba, Japan

**Keywords:** small intestine, microbiota, endurance athletes, recovery, endurance performance

## Abstract

The special issue “Physiological Aspects of Marathon Running” in “Frontiers in Physiology” aims to evoke attentions to this field. In this minireview, the two guest editors of the special issue introduce their interest in the gastrointestinal (GI) functions with endurance running and gut microbiota on marathon running. Following the introduction, the first part summarized research examining exercise-induced GI damage and methods to mitigate the damage. The latter part summarized the influence of exercise on gut microbiota, and the gut microbiota on endurance performance. From the brief overview above, this minireview synthesizes current knowledge and identifies critical topics for future studies for optimizing GI function and the gut microbiota in endurance athletes.

## 1 Introduction

There has been extensive research on exercise training, recovery, and performance in endurance athletes focusing on skeletal muscle, respiratory and circulatory function, vascular function, and energy metabolism. However, recent research has emphasized the role of the gastrointestinal (GI) tract, with a particular focus on “GI damage” and “gut microbiota.” Keeffe et al. reported that marathon runners experienced bowel movements (35%) and diarrhea (19%) after running, occasionally preventing them from running (Keeffe et al., 1984). Runners often have more abnormalities in the lower digestive tract than in the upper digestive tract (Peters et al., 1999). Presently, it is believed that 30%–70% of athletes experience GI problems (de Oliveira and Burini, 2009; Coleman, 2019), although one study suggests that the prevalence is not that high (ter Steege et al., 2008). Therefore, for endurance athletes, the digestive tract is a vital organ for good training and athletic performance.

The GI tract is responsible for the digestion and absorption of ingested nutrients and acts as a protective barrier against bacterial translocation. Nevertheless, strenuous exercise (e.g., prolonged exercise) decreases GI function (Chantler et al., 2021). Prolonged exercise increases both GI (small intestinal) damage and the permeability of the intestinal barrier, which allows the translocation of bacteria and its unfavorable metabolites (i.e., endotoxin) into the systemic circulation, induces systemic inflammatory and anti-inflammatory cytokine secretion, increases body temperature, aggravates subjective GI symptom (such as gastric distention and discomfort) (Chantler et al., 2021). Furthermore, increased levels of intestinal fatty acid-binding protein (I-FABP) in the blood, an indirect indicator of small intestinal damage, decrease the rate of digestion and absorption of ingested nutrients (van Wijck et al., 2013). Considering that muscle protein synthesis and muscle glycogen resynthesis are maximized in the early phase of postexercise period (Koopman et al., 2007; Beelen et al., 2010; Burke et al., 2017), the increase in exercise-induced GI damage can delay the ingested nutrients to the muscle, which might negatively impact post-exercise recovery. Therefore, reducing GI damage following endurance exercise is important in terms of improving endurance exercise performance and promoting post-exercise recovery.

Several hundred species comprising 100 trillion bacteria are estimated to live in the human gut. Endotoxins (Simons and Kennedy, 2004) and an imbalance in the composition of the gut microbiota (dysbiosis) (Bonomini-Gnutzmann et al., 2022; Patel et al., 2024) have been implicated in lower GI symptoms in endurance athletes. One study reported that the difference in microbiota composition between rugby players and controls led to the recognition of the impact of exercise on microbiota (Clarke et al., 2014). Since then, several studies have been conducted and a systematic review has been published on the effects of physical activity (Aya et al., 2021) and endurance exercise (Bonomini-Gnutzmann et al., 2022) on gut microbiota. Nonetheless, discrepancies exist in those studies. For instance, *Faecalibacterium* has been reported to be less abundant in marathon runners than in sedentary controls (Kulecka et al., 2020); however, it has also been reported to be more abundant in female endurance runners than in age-matched controls (Morishima et al., 2021). Therefore, to clarify this discrepancy, this review organizes the studies on gut microbiota in terms of changes induced by a single exercise or race and those induced by repeated training, with a comparison between endurance athletes and nonathletes, and focuses on the types of microbiota composition. Finally, directions for future studies are suggested.

The purpose of this minireview is to introduce the changes in GI function with endurance exercise and role of gut microbiota in marathon running. This is one of the topics that we, the two guest editors of the special issue “Physiological Aspects of Marathon Running” in “Frontiers in Physiology”, would like to explore in the issue. The literature reviewed in this minireview was published or searched using PubMed by the 24th of December 2024.

## 2 Gastrointestinal function with endurance exercise

### 2.1 Endurance exercise and GI damage

Endurance exercise, particularly prolonged and/or high-intensity endurance exercise, induces significant GI damage. During exercise, blood flow is preferentially distributed to the skeletal muscle, which temporarily reduces GI blood flow (van Wijck et al., 2011). This hypoperfusion in the GI tract increases tissue hypoxia and oxidative stress, resulting in intestinal cell damage and alterations in endothelial tight junctions. In the past few years, knowledge has accumulated on the factors that elevate GI damage associated with endurance exercise. Regarding exercise intensity, higher exercise intensity increases muscle blood flow, leading to lower GI blood flow as well as higher GI damage (Edwards et al., 2021). Conversely, differences in exercise duration may exert a smaller effect on GI symptoms and GI damage (Edwards et al., 2021; Gaskell et al., 2023). Focusing on the differences in exercise modalities, cycling or running endurance exercise for 45 min at relatively identical intensity (70% of ${\dot{V}O}_{2\max}$ ) resulted in greater increases in blood I-FABP levels for cycling exercise than for running exercise (Edwards et al., 2021). However, RPE and HR during exercise in that study was significantly higher in the cycling trial. In contrast, GI symptoms and GI damage after exercise in the heat were similar between cycling exercise (55% of maximal aerobic power) and running exercise (55% of ${\dot{V}O}_{2\max}$ ) when the RPE, HR, and rectal temperature during exercise were matched between the two trials (Costa et al., 2022). Further research is necessary to explore the effects of different exercise modalities on exercise-induced GI damage.

The effect of thermoregulation during exercise, involving dehydration and heat stress, has been well documented as a factor increasing exercise-induced GI damage. Costa et al. (2019) demonstrated that hypohydration (3.1% of BW loss) during a 2-h run significantly increased blood I-FABP concentrations associated with exercise compared with euhydration (0.6% of BW loss). They speculated that increased dehydration significantly reduced GI blood flow followed by increased GI damage. Heat stress is also a major factor that elevates GI damage (Snipe et al., 2018a; 2018b; Osborne et al., 2019b; Wallett et al., 2021; Sumi et al., 2024). Exercise in the heat increases skin blood flow and sweat volume to promote heat dissipation from the skin compared with exercise in a thermoneutral environment, which induces a greater reduction in GI blood flow and elevated GI damage. Therefore, elevated core temperature and dehydration (or hypohydration) are probably the most influential factors that increase GI damage. Even under mild heat stress conditions (<30°C), prolonged exercise such as a full marathon would cause significant GI damage due to dehydration and elevated core temperature. In fact, a large number of runners complain of GI symptoms during full marathon races (Kelly et al., 2023).

In addition to heat stress, a hypoxic environment elevates exercise-induced GI damage. Endurance running in hypobaric hypoxia or normobaric hypoxia (fraction of inspired oxygen: 13.5%–13.8%) elevates GI damage and systemic inflammatory responses compared with the same exercise (i.e., same running velocity or same pedaling work load) in normoxia (Hill et al., 2020; McKenna et al., 2022). Exercise in hypoxia reduces oxygen saturation in the working muscle due to lower blood oxygen saturation, which results in higher muscle blood flow than exercise in normoxia (Casey and Joyner, 2012; Joyner et al., 2014). Therefore, GI blood flow may be considerably reduced and GI damage may be elevated after exercise in hypoxia. Nevertheless, the blood I-FABP response after endurance running was similar between hypoxia and normoxia when exercise intensity was relatively matched (i.e., same % of ${\dot{V}O}_{2\max}$ ) between hypoxia and normoxia (Nomura et al., 2024).

Various factors that elevate exercise-induced GI damage have been demonstrated. Perhaps a greater reduction in GI blood flow, especially due to increased body water loss and redistribution of blood flow to the peripheral tissues (i.e., skin and working muscle), increases exercise-induced GI damage. Conversely, considering that elevated GI damage negatively affects performance and promotes recovery after exercise, it is necessary to develop strategies for reducing exercise-induced GI damage.

### 2.2 Effective strategy reducing exercise-induced GI damage

GI damage following endurance exercise induces greater inflammatory response, GI symptoms and lower absorbed and digestive capacity of macronutrients. These responses would have a negative impact on performance and recovery. For example, lower absorbed and digestive capacity of ingested macronutrient may negatively affect for recovery of nutritional aspect, considering that muscle protein synthesis and muscle glycogen resynthesis rate are maximally stimulated during the early phase of post-exercise period (Koopman et al., 2007; Beelen et al., 2010; Burke et al., 2017).

Exercise-induced GI damage can be alleviated by using various procedures, particularly macronutrient supplementation that has been widely reported. March et al. (2017), March et al. (2019) showed that 2 weeks of bovine colostrum intake improved cellular protection function via an increase in HSP70 levels, which contributed toward reducing exercise-induced increases in blood I-FABP levels. In contrast, a shorter (7 days) supplemental period (Morrison et al., 2014) or severe heat stress (e.g., >40°C) during exercise (McKenna et al., 2017) was not likely to mitigate exercise-induced GI damage following bovine colostrum supplementation. Studies have also reported the effects of a single intake of glutamine before exercise (Pugh et al., 2017; Osborne et al., 2019a) and 4 weeks of probiotic intake (Pugh et al., 2019; 2020; Mooren et al., 2020), although the results were inconsistent. Regarding macronutrients, ingestion of a drink containing carbohydrates alone or a mixture of carbohydrates and other nutrients (protein and pectin alginate) before and during exercise reduced exercise-induced GI damage (Snipe et al., 2017; Flood et al., 2020). It has been considered that the ingestion of nutrients containing sufficient calories alleviates GI damage by increasing GI blood flow. In particular, the two abovementioned studies investigated the response to exercise in the heat. Considering that heat stress strongly affects exercise-induced GI damage, glucose ingestion may be a valuable strategy to mitigate exercise-induced GI damage in the heat. However, glucose intake in the gel form may not exert a positive effect (Sessions et al., 2016).

The effects of ingesting water at different temperatures on exercise-induced GI damage in the heat have also been examined (Snipe and Costa, 2018). Skin blood flow during exercise increases in the heat due to heat dissipation, and subsequently GI blood flow reduces. Therefore, ingestion of lower temperature water may reduce the increase in core temperature during exercise, which may contribute to a reduction in GI damage due to the maintenance of GI blood flow (decrease in skin blood flow). Snipe and Costa (2018) demonstrated that COOL (7°C) and COLD (0°C) water ingestion did not significantly reduce the increase in blood I-FABP levels, whereas the increase in rectal temperature (Δ) was lower in the COOL (1.7°C ± 0.4°C) and COLD (1.6°C ± 0.4°C) conditions than in the TEMP (2.0°C ± 0.5°C) condition. They speculated that water ingestion at a temperature of 0°C–7°C was insufficient to reduce heat strain and therefore did not reduce exercise-induced GI damage in the heat. Another study also examined the effects of ice-slurry ingestion before and during exercise but found no reduction in exercise-induced increases in blood I-FABP levels in the heat (Alhadad et al., 2023).

Conversely, Zadow et al. (2020) reported the effects of wearing a compression garment (socks) during endurance exercise on exercise-induced GI damage. Wearing a compression garment on the lower legs increases venous return, which may cause maintenance of blood flow in the central region (e.g., the GI tract). They showed that wearing compression socks with 25 mm Hg during a marathon race decreased the exercise-induced increases in blood I-FABP compared with that in the non-wearing group. However, no significant difference was observed in the marathon race time between the two groups (Zadow et al., 2020).

Most previous studies that investigated exercise-induced GI damage (elevations in blood I-FABP levels) have focused on GI permeability, systemic inflammatory response, and GI symptoms with endurance exercise. In contrast, exercise-induced elevations of blood I-FABP levels are associated with delayed digestion and absorption rates following macronutrient ingestion (van Wijck et al., 2013). Nonetheless, a few studies have explored the relationship between exercise-induced GI damage and the rate of digestion and absorption of ingested nutrients, but the evidence is lacking. Therefore, further studies are required to determine the nutritional aspects of recovery (e.g., muscle glycogen resynthesis and muscle protein synthesis) following exercise with a developing strategy to mitigate exercise-induced GI damage.

## 3 Roles of gut microbiota on endurance running

### 3.1 Gut microbiota of endurance athletes

Here, we discuss the literature on the gut microbiota of endurance athletes in terms of changes induced by a single exercise or race and those induced by repeated training, with a comparison of endurance athletes and focusing on the types of microbiota composition.

Regarding the changes in gut microbiota induced by a single bout of exercise and a single-stage race, Tabone et al. investigated the effect of short duration of high-intensity exercise (Tabone et al., 2021). In their study, 40 male endurance cross-country athletes performed an incremental treadmill run with a slope of 1% at a speed of 10 km/h, with increments of 0.3 km/h every 30 s until exhaustion followed by a 1-km run on an athletics track at the maximum speed. Their feces were collected before and within 4 h without diet intake postexercise. Results showed that exercise increased the abundance of *Romboutsia*, *Ruminococcaceae* UCG-005, *Escherichia coli* TOP498, and *Blautia* and decreased the abundance of *Ruminiclostridium* nine and *Clostridium phoceensis* (Tabone et al., 2021). Another study reported that a half-marathon race increased the abundance of *Ruminococcus bicirculans* and *Collinsella aerofaciens* and decreased that of six species, including *Bacteroides coprophilus*, in the feces of amateur athletes (Zhao et al., 2018). A full marathon race was found to increase the abundance of *Veillonella* (Scheiman et al., 2019). Furthermore, a world-class male ultramarathon runner completed the Western States Endurance Race, a 163-km mountain footrace with ∼5,486 m of climbing and ∼7,010 m of descending, in approximately 16 h. His gut microbiota revealed that the ultramarathon increased the abundance of *Haemophilus*, *Streptococcus*, and *Veillonella* and decreased that of *Alloprevotella* and *Faecalibacterium* (Grosicki et al., 2019). Sato and Suzuki (2022) reported the effect of a longer duration, ∼44 h, of a 96-km ultramarathon (up 8,062 m, down 6,983 km). The feces of nine male runners revealed a significant decrease in the abundance of four butyrate-producing species, including *Faecalibacterium prausnitzii*, and an increase in the abundance of ten species, including *Collinsella aerofaciens*, whereas no significant change was observed in that of *Veillonella* (Sato and Suzuki, 2022). Therefore, irrespective of whether a single exercise or race, the duration can exert changes in the gut microbiota.

Because endurance athletes habitually engage in endurance exercise, the changes caused by repeated aerobic training have been investigated. For instance, Allen et al. examined the impact of 6 weeks of supervised, endurance-based exercise training (3 days/week) on the gut microbiota of lean and obese sedentary subjects (Allen et al., 2018).They found an increase in the abundance of *Faecalibacterium* and *Lachnospira* and a decrease in that of *Bacteroides* in the gut microbiota of lean subjects. Furthermore, they observed increases in the fecal concentrations of SCFAs (acetate, propionate, and butyrate) (Allen et al., 2018). Another study investigated the effect of 3 weeks of high-volume training in highly trained middle-distance runners and reported a significant increase in the abundance of *Ruminococcus callidus* and decreases in the abundance of *Streptococcus parasanguinis* and *Haemophilus parainfluenzae* (Craven et al., 2022). Therefore, there is still a limitation of studies on the changes in gut microbiota due to repeated exercise interventions, with inconsistent results.

As habitual endurance exercise influences the gut microbiota, the difference in gut microbiota has been investigated by comparing endurance athletes with nonathletes. For instance, Kulecka et al. compared marathon runners, cross-country skiers, and sedentary healthy controls and observed significant differences in the abundances of 16 taxa in marathon runners and 5 taxa in cross-country skiers compared with that in controls (Kulecka et al., 2020). Both marathon runners and cross-country skiers showed significantly higher abundance of *Eggerthellacea*_uncultured and *Prevotella*_9 and lower abundance of *Bacteroides* than the controls. Furthermore, marathon runners showed higher abundance of *Haemophilus* and *Veillonella* and lower abundance of *Blautia* and *Faecalibacterium* than the controls. Significant differences in abundance were also detected in the family *Coriobacteriaceae* and two genera belonging to the family *Lachnospiraceae* between marathon runners and cross-country skiers (Kulecka et al., 2020). *Veillonella* has also been reported to be more prevalent among marathon runners than among nonrunners (Scheiman et al., 2019). In addition, Morishima et al. reported that female Japanese elite endurance runners had higher abundance of *Faecalibacterium* and *Lachnospira* and lower abundance of *Haemophilus* than healthy volunteers, with 13 other taxa exhibiting significant differences in abundance (Morishima et al., 2021). In another study, male Japanese college long-distance runners showed higher abundance of *Bacteroides*, *Prevotella*, and *Lachnospira* than nonathletes, with six other genera showing significant differences in abundance (Morita et al., 2023). Therefore, the gut microbiota of endurance athletes differs between marathon runners and cross-country skiers, and even if we limit the discussion to endurance runners, we can still notice the discrepancies depending on the report.

Another important aspect is that the human gut microbiota can be classified into several categories, viz., enterotypes. Partitioning around medoids clustering of the human gut microbiota reveals three distinct enterotypes, B-, P-, and R-types, whose major contributors are *Bacteroides*, *Prevotella*, and *Ruminococcus*, respectively (Arumugam et al., 2011). The gut microbiota of Asian people can be classified into two major types, the *Prevotella*-rich P-type and the *Bifidobacterium*- and *Bacteroides*-rich BB-types (Nakayama et al., 2015). Nevertheless, there has been no clarification on what causes the enterotype, including its possible involvement in the living environment, lifestyle including dietary habits, and genotype. Enterotypes can affect the change induced by exercise; however, only a few studies have examined this aspect. Only the study by Sato and Suzuki (2022) reported that their participants’ enterotypes were classified as B-type (n = 6) and P-type (n = 3); however, the changes in the abundance of *Faecalibacterium prausnitzii* and *Collinsella aerofaciens* occurred unanimously irrespective of the enterotype (Sato and Suzuki, 2022). Enterotype may be involved in the discrepancies observed in several studies. Therefore, it is necessary to increase the focus on the role of enterotype in the gut microbiota of endurance athletes.

### 3.2 Gut microbiota and endurance performance

Intestinal bacteria ferment dietary fiber into SCFAs, which exert beneficial effects on colon health (Jenkins et al., 1995; Mortensen and Clausen, 1996) and constitute 40%–50% of the available energy of carbohydrates (Cummings and Macfarlane, 1997). Therefore, it is believed that SCFAs produced in the gut subsequently function as important energy substrates for both liver and muscle cells, thereby improving overall endurance (Mach and Fuster-Botella, 2017). Recently, there have been two important studies that demonstrate this phenomenon.

The first impact was the study by Scheiman et al.who hypothesized that *Veillonella* would be beneficial for marathon running because its abundance increases after a full marathon race, showing higher abundance in marathon runners than in nonrunners (Scheiman et al., 2019). They inoculated mice with *Veillonella atypica* isolated from a runner and found that the mice ran longer on the treadmill until exhaustion than the control mice inoculated with *Lactobacillus bulgaricus*. As *Veillonella* converts lactic acid to SCFAs, primarily propionate, they injected propionate into the colorectum of mice and confirmed the prolongation of the time required to complete the treadmill run. Based on these observations, they suggested that *Veillonella* in the colon converts lactic acid produced in the blood during exercise into SCFAs, primarily propionate, and that SCFAs improve endurance performance.

Then came the second impact with the study by Morita et al. who observed that Japanese male long-distance runners had a high abundance of *Bacteroides*, especially *Bacteroides uniformis*, and this abundance correlated with the 3000-m race times (Morita et al., 2023). They then conducted a randomized controlled trial to show that 9 weeks of α-cyclodextrin supplementation increased the abundance of *Bacteroides uniformis* and shortened 10-km cycling times in Japanese adult males. Moreover, when administered to mice, it increased the swimming time, inhibited the exercise-induced decreases in cecal SCFA levels (acetate, butyrate, propionate, and valerate), and increased the expression of carnitine palmitoyltransferase 1 and phosphoenolpyruvate carboxykinase 1, which are involved in hepatic beta-oxidation and gluconeogenesis, respectively. These findings suggest that *Bacteroides uniformis* produces propionate and acetate in the intestinal tract that stimulates hepatic gluconeogenesis, thereby improving endurance.

The two abovementioned studies (Scheiman et al., 2019; Morita et al., 2023) revealed different bacteria from the feces of endurance runners, viz., *Veillonella atypica* and *Bacterides uniformis*, respectively; however, both studies demonstrated that the production of SCFAs, primarily propionate, in the intestinal tract improved endurance performance. These findings suggest that the metabolic function/pathway of the two species contributes to their effects on endurance performance. Nevertheless, it remains unclear whether propionate acts as an energy fuel or as a first messenger to exert physiological response. If it acts as an energy fuel, it is necessary to clarify the reason why it is advantageous over acetate, which is more abundant in the gut, and if it acts as a signal, it is important to clarify the direct effector and the signaling pathway.

Conversely, from the perspective that SCFAs produced by gut microbiota are beneficial to health, there have also been attempts to use SCFAs as ergogenic supplements. When healthy people consumed sodium propionate, their resting energy expenditure showed greater increases than when they consumed sodium chloride (Chambers et al., 2018). Moreover, when healthy men ingested sodium acetate (4 mmol/kg) and rode a cycle ergometer for 120 min, the preexercise acetate concentration in the blood was 13 times higher than when the same amount of NaHCO_3_ was administered, and there were significant changes in lipid and carbohydrate oxidation, whereas no significant difference in energy expenditure during the cycling task at ∼50% VO_2_peak was reported at any time point (Smith et al., 2013). However, the mean energy expenditure measured every 15 min from 15 to 120 min was greater with acetate supplementation than that in the control at all measurement points (Smith et al., 2013). Therefore, if the area under the curve had been compared, the energy expenditure during exercise could have been significantly greater with acetate supplementation. Although there are several other studies using animals and cells, only a few have demonstrated the effects on exercise capacity in humans, and there exists a need to explore the direct effects of SCFAs on exercise capacity (Ong et al., 2023).

Probiotics have also been reported to improve athletic performance. Single strains include species belonging to *Bifidobacterium* and *Lactobacillus*, and multistrain combinations may include *Enterococcus faecium*, *Bacillus subtilis*, and *Streptococcus thermophilus* in addition to strains belonging to *Bifidobacterium* and *Lactobacillus* (Santibañez-Gutierrez et al., 2022). A meta-analysis found that probiotic supplementation may improve performance in studies of populations that have undergone training with a predominance of aerobic metabolism (Santibañez-Gutierrez et al., 2022). Moreover, a recent systematic review revealed 13 studies that investigated the impact of probiotic supplementation on the performance of athletes, of which 7 were related to endurance athletes (Di Dio et al., 2023). The present review does not mention individual studies summarized in the abovementioned reviews because both reviews have excellent evidence tables that should be consulted.

Nonetheless, most studies on probiotics used specific subspecies of some species and claimed that the special compounds in that strain exert special effects. This may be because specific subspecies are commercial products. In the 2022 meta-analysis, the effects of probiotics were not separated by strain, but rather a meta-analysis of all targeted studies was used as the effect of probiotics. However, although there is some evidence reporting a lack of effect, there is no single study reporting negative results for probiotics (Santibañez-Gutierrez et al., 2022). This situation may reflect the fact that the motivation for reporting the effects of specific strains is to demonstrate that they are effective, i.e., there exists a publication bias.

Despite the possible publication bias in studies on probiotics, it is certainly possible that certain species exert physiological effects through the action of specific products rather than metabolites. This phenomenon is illustrated by the fact that certain pathogenic bacteria can cause specific diseases through their specific toxins. Nevertheless, although the pathogenic mechanisms of pathogenic strains have generally been elucidated, most mechanisms underlying the beneficial effects of probiotics have not been elucidated. For instance, a heat-killed preparation of a certain *Lactococcus* strain exerted the same effect on the hematopoietic system (Suzuki et al., 2022b; 2022c; Takaragawa et al., 2022) as that observed with the live preparation (Kimoto-Nira et al., 2014), which strongly suggests the involvement of a substance other than metabolites; however, the underlying mechanism has not been investigated. Nevertheless, research on *Faecalibacterium prausnitzii*, which is being touted as a next-generation probiotic, has fairly progressed (He et al., 2021). An increase in the abundance of *F. prausnitzii* was found to affect the peripheral lymphocyte subset (Jinnouchi et al., 2021) and cytokines (Suzuki et al., 2022a) of healthy individuals. As a mechanism that can explain these effects on the immune system, it has been demonstrated that seven peptides derived from a single microbial anti-inflammatory molecule known as “MAM” activate the immune system (Quévrain et al., 2016). Therefore, if a specific strain is demonstrated to improve endurance performance, it is also necessary to elucidate the effects specific to that strain, other than common metabolites, at the molecular level.

Considering our discussion of the possible contribution of gut microbiota to endurance performance, the difficulty of describing a clear strategy at this time has become apparent. Nonetheless, the topics for further research have also become clear. Although there are studies emphasizing the importance of propionate (Chambers et al., 2018; Scheiman et al., 2019; Morita et al., 2023), the most abundant among SCFAs is acetate, with studies illustrating the effects of acetate-producing *Bifidobacterium* (Lin et al., 2020) and acetate itself (Smith et al., 2013). Therefore, it is important to clarify 1) which SCFA is effective (or whether all of them are similar) and 2) whether the mechanism of action is an energy source or promotes metabolic regulation. Furthermore, if an effect on a specific genus, species or subspecies is demonstrated, the mechanism should be elucidated at the molecular level. Because there are several hundred species comprising 100 trillion bacteria living in the gut and the ecosystem can be classified into several enterotypes, it is also essential to clarify the impact of enterotypes on the action of metabolites or bacterial substances.

As shown in Table 1, we will suggest significant future studies in this research field. These studies would provide valuable information on endurance performance and recovery in endurance athletes.

**TABLE 1:** Topics for future study to understand the role of the gastrointestinal tract and gut microbiota and to develop strategies to improve endurance performance.

The following measurements should be evaluate simultaneously with changes in exercise-induced GI damage
• Appearance rate of ingested macronutrients (e.g., glucose and amino acids) into the blood circulation
• Muscle glycogen recovery and muscle protein resynthesis
• Energy intake and appetite
Gut microbiota
• Short-chain fatty acids: an energy source and/or a signal messenger to modify physiological function?
• Role of bacteria: metabolic pathway and metabolite or species-specific substance?
• Enterotype: Does it influence the change induced by exercise? This topic should be studied while controlling for the possible confounding factors such as dietary habits, ethnicity, and living environment

## 4 Conclusion

This review emphasizes the importance of maintaining GI function and gut microbiota in endurance athletes. Although strategies to reduce exercise-induced GI damage have been identified, their effectiveness varies with exercise conditions and environmental factors. Recent studies have shown that specific bacterial species, particularly those producing SCFAs, may improve endurance performance. However, several critical questions remain unanswered, including which SCFAs are most effective for performance improvement and whether they function primarily as energy sources or a signal to metabolic adaptations. Future research should focus on clarifying the molecular mechanisms through which specific bacterial species affect athletic performance and understanding how different enterotypes affect these relationships. As our understanding of these area advances, it would enable the development of more effective strategies for optimizing GI function and utilizing gut microbiota to improve endurance performance.
